# Mouse Model Reveals the Role of RERE in Cerebellar Foliation and the Migration and Maturation of Purkinje Cells

**DOI:** 10.1371/journal.pone.0087518

**Published:** 2014-01-23

**Authors:** Bum Jun Kim, Daryl A. Scott

**Affiliations:** 1 Department of Molecular and Human Genetics, Baylor College of Medicine, Houston, Texas, United States of America; 2 Department of Molecular Physiology and Biophysics, Baylor College of Medicine, Houston, Texas, United States of America; Tokyo Medical and Dental University, Japan

## Abstract

Nuclear receptors and their coregulators play a critical role in brain development by regulating the spatiotemporal expression of their target genes. The arginine-glutamic acid dipeptide repeats gene (*Rere*) encodes a nuclear receptor coregulator previously known as Atrophin 2. In the developing cerebellum, RERE is expressed in the molecular layer, the Purkinje cell layer and the granule cell layer but not in granule cell precursors. To study RERE's role in cerebellar development, we used RERE-deficient embryos bearing a null allele (*om*) and a hypomorphic allele (*eyes3*) of *Rere* (*Rere* ^om/eyes3^). In contrast to wild-type embryos, formation of the principal fissures in these RERE-deficient embryos was delayed and the proliferative activity of granule cell precursors (GCPs) was reduced at E18.5. This reduction in proliferation was accompanied by a decrease in the expression of sonic hedgehog (SHH), which is secreted from Purkinje cells and is required for normal GCP proliferation. The maturation and migration of Purkinje cells in *Rere* ^om/eyes3^ embryos was also delayed with decreased numbers of post-migratory Purkinje cells in the cerebellum. During the postnatal period, RERE depletion caused incomplete division of lobules I/II and III due to truncated development of the precentral fissure in the cerebellar vermis, abnormal development of lobule crus I and lobule crus II in the cerebellar hemispheres due to attenuation of the intercrural fissure, and decreased levels of Purkinje cell dendritic branching. We conclude that RERE-deficiency leads to delayed development of the principal fissures and delayed maturation and migration of Purkinje cells during prenatal cerebellar development and abnormal cerebellar foliation and Purkinje cell maturation during postnatal cerebellar development.

## Introduction

Cerebellar foliation is a highly conserved feature of the mammalian brain with all mammals having at least ten folia along the anterior-posterior (AP) axis of the cerebellar vermis [1]–[3]. Foliation contributes to expanded surface area, permitting the mammalian cerebellum to accommodate more cells and allowing for increased numbers of sophisticated neural circuits [4]. Some types of neural circuits are located exclusively within specific folia. For example, spinocerebellar mossy fibers project only to the anterior lobules I-V and posterior lobules VIII/IX in the cerebellar vermis of mice [5]–[7]. Since the cerebellum provides a platform for innervations of neural circuits carrying sensory-motor information, it is not surprising that processes that disrupt foliation are frequently associated with abnormal motor behaviors including ataxia and impaired motor coordination [1], [5], [6], [8], [9].

In mice, the cerebellar primordium is formed between embryonic days 9 (E9) and 12 (E12) and is transformed into a cylindrical structure by E15.5 [10]. After birth, the cerebellum grows rapidly with robust expansion of granule cells and begins to form three landmark regions—the central vermis, the lateral hemispheres, and the lateral most flocculi and paraflocculi. A specific set of fissures and folia is found in the vermis and the hemispheres at adulthood. Mice have at least ten lobules in the vermis—lobules I through X—and four lobules in the hemispheres—the simple lobule, crus I of the ansiform lobule, crus II of the ansiform lobule and the paramedian lobule—along the anterior-posterior (AP) axis [1]–[3], [11].

Foliation of the principal fissures initiates in the vermis area around E17.5. By E18.5, the five cardinal lobes (anterobasal, anterodorsal, central, posterior, and inferior lobes) and the four principal fissures (preculminate, primary, secondary, and posterolateral fissures) can be identified [1], [12]. After birth, the cardinal lobes are gradually divided by additional fissures. The anterobasal lobe is the first folia to be divided at P0 and forms lobules I through III [3], [13]. Lobules VII and VIII are developed from the central lobe by P1. Lobule VI is demarcated from lobule VII by P3. By P5, the anterodorsal lobe is transformed into lobules IV and V. The posterior lobe becomes lobule IX and the inferior lobe corresponds to lobule X [12]. Development of all ten folia along the AP axis of the vermis is completed around P7 in most mouse strains.

Two dynamic cellular events, the proliferation of the granule cell precursors (GCPs) and the anchoring of Purkinje cells, drive folial development [1], [14]–[17]. At E17.5, the differential proliferation rate of GCPs induces thickening of the external granule cell layer (EGL) at sites that will ultimately form the bases of the principal fissures. This thickening is accompanied by invagination of the EGL and folding of the Purkinje cell layer (PCL). Sudarov and Joyner defined the base of each developing fissure as an anchoring center [12]. Anchoring centers acquire a distinct cytoarchitecture with GCPs located at anchoring centers becoming distinctly elongated along the axis of the forming fissure. Bergmann glia cells located at the base of the immature fissures radiate their fibers towards the anchoring center. This is in contrast to most areas of the cerebellum, where fibers from Bergmann glial are oriented parallel to each other and project perpendicular to the surface of the EGL [12]. In addition, Ma et al. showed that Ric-8a, a guanine nucleotide exchange factor in the G-protein-coupled receptor pathway, is required to maintain adhesion between Bergmann glia and the basement membrane and that this adhesion is required for complete fissure formation during cerebellar development [18].

It is well established that sonic hedgehog (SHH) signaling regulates the expansion of GCPs during cerebellar development [15], [17]. Since GCP proliferation is a critical factor in folial development, it is not surprising that alterations in SHH signaling pathway genes cause cerebellar hypoplasia and abnormal cerebellar foliation [14], [16], [19], [20]. SHH is expressed in the Purkinje cells of the cerebellum [15], [17]. Thus, crosstalk between GCPs and Purkinje cells plays a critical role in normal foliation.

The arginine-glutamic acid dipeptide (RE) repeats gene (*Rere*) is named after the dipeptide repeats found in RERE's carboxyl terminal. RERE was previously known as Atrophin 2 (ATR2) based on the similarities seen between RERE and Atrophin 1 (ATR1). In particular, the C-terminal region of RERE is highly homologous to that of ATR1. However, RERE has unique BAH, ELM2, SANT, and GATA domains in its N-terminal region which are thought to play a role in protein/protein interactions, chromatin remodeling and DNA binding [21]. It has been proposed that RERE acts as a nuclear receptor coregulator through physical interaction with members of nuclear receptor subfamily 2 (NR2) such as NR2F2 (COUP-TFII) and NR2E1 (TLX) [22], [23]. In addition, RERE also has been shown to recruit histone deacetylase 1/2 (HDAC1/2) *in vitro* and in mouse embryos [23], [24]. This function requires expression of RERE's N-terminal region.

The role of RERE during embryonic development has been explored using mouse models. Zoltewicz et al. identified a mouse line carrying an N-ethyl-N-nitrosourea (ENU) induced splice junction mutation in *Rere* (c.396+2T>A) which they named *openmind* (*om*) based on the open neural tube defects seen in embryos that were homozygous for this mutation [24]. This mutation disrupts the splice donor site of *Rere*'s second coding exon causing skipping of this exon, a shift in the reading frame and induction of a premature stop codon. Whole-mount *in situ* hybridization revealed that *Rere* mRNA was virtually undetectable in *Rere* ^om/om^ embryos, suggesting that this mutation was a null allele [24]. We subsequently demonstrated that *Rere* ^om/om^ embryos also have undetectable levels of RERE protein by western blot [25].

Mice that are heterozygous for the *om* allele (*Rere* ^+/om^) are viable and fertile. In contrast, *Rere* ^om/om^ embryos have heart looping defects and die with signs of cardiac failure between E9.5 and E11.5 [24]. These embryos also have fusion of the telencephalic vesicles, defects of the optic vesicles and failure of anterior neural tube closure [24]. These data suggest that RERE plays a critical role in early development of the central nervous system. However, *Rere* ^om/om^ embryos cannot be effectively used to determine the role of RERE in the development of the cerebellum whose patterning and maturation begin during the latter stages of embryonic development and are not completed until the postnatal period.

In this study we used RERE-deficient embryos and mice bearing the *om* null allele and a hypomorphic *Rere* allele, *eyes3* (c.578T>C, p.Val193Ala) to study the role of RERE during cerebellar development. The *eyes3* allele was identified in an ENU-based screen and causes its deleterious effects on development by reducing RERE function rather than decreasing RERE protein expression [25]. Unlike *Rere* ^om/om^ embryos which die around E9.5, *Rere* ^om/eyes3^ mice can be recovered in Mendelian ratios at birth [25]. Although the majority of these mice die in the perinatal period due to cardiac and renal defects, some survive into adulthood making it possible to determine the effect of RERE depletion on all stages of cerebellar development.

## Materials and Methods

### Ethics statement

All experiments using mouse models were conducted in accordance with the recommendations in the Guide for the Care and Use of Laboratory Animals of the National Institutes of Health. The associated protocols were approved by the Institutional Animal Care and Use Committee of Baylor College of Medicine (Animal Welfare Assurance #A3832-01).

All efforts were made to minimize suffering. Experiments that were likely to induce discomfort, distress, pain, or injury, or required the use of restraining devices were performed on mice that had been properly anesthetized by inhalation of isoflurane in an appropriate enclosure. Euthanasia was carried out using methods consistent with the recommendations of the Panel of Euthanasia of the American Veterinary Medical Association and included inhalation of carbon dioxide or an overdose of an inhaled anesthetic, such as isoflurane, in an appropriate enclosure.

### Mouse strains

C57BL/6 embryos and mice were used to define the expression pattern of RERE in the cerebellum. The generation of the *om* and *eyes3* alleles of *Rere* were described previously [24], [25]. Experiments using these alleles were conducted on a mixed B6/129S6 background.

### Preparation of paraffin embedded tissue sections and tissue staining

Tissues were removed by dissection and fixed with Buffered Formalde-Fresh solution (Fisher Scientific, Pittsburgh, PA, USA) for 1 day at 4°C. After washing with phosphate buffered saline solution (PBS), tissues were dehydrated in ethanol and embedded in paraffin. Paraffin embedded tissue blocks were sectioned at 6 µm with an RM2155 microtome (Fisher Scientific). Nissl staining was used for histological analyses of the cerebellum.

### Immunohistochemical staining

After deparaffinization, cerebellar tissue sections were blocked with 1X PBS containing 1% bovine serum albumin and 5% normal donkey serum for 1 hour at room temperature. Sections were then incubated with anti-RERE (sc-98415, 1∶100; Santa Cruz Biotechnology, Santa Cruz, CA, USA), rabbit polyclonal anti-calbindin (AB1778, 1∶200; Millipore, Billerica, MA, USA), mouse monoclonal anti-calbindin (C9848, 1∶100; Sigma, St. Louis, MO, USA)—used only for double labeling experiments with anti-RERE—anti-Phospho-Histone H3 (pHH3) (#9701, 1∶200; Cell Signaling, Danvers, MA, USA), anti-Pax6 (PAX6, 1∶200; Developmental Studies Hybridoma Bank (DSHB), Iowa, IO, USA), anti-NR2F2 (ab41859, 1∶1000; abcam, Cambridge, MA, USA), or anti-Cleaved Caspase-3 (#9664, 1∶200; Cell Signaling, Danvers, MA, USA) antibodies diluted in the same blocking solution (1% BSA and 5% normal donkey serum in 1X PBS) overnight at 4°C. Information about the specificity and previous use of each of the primary antibodies used in this study is summarized in Table S1.

After washing with 1X PBS, the sections were incubated with biotin conjugated anti-rabbit IgG or biotin conjugated anti-mouse IgG (Jackson ImmunoResearch, West Grove, PA, USA). Immunoreactivity of each antibody was visualized using either a 3,3′-diaminobenzidine (DAB) substrate kit (Vector Laboratories, Burlingame, CA, USA) or a tyramide signal amplification (TSA) kit (Invitrogen, Grand Island, NY, USA) containing Alexa Fluor 488 dye or Alexa Fluor 568 dye for fluorescent labeling per manufacturer's instructions. Images were acquired on a Zeiss Axioplan microscope equipped with an AxioCam digital camera and imaging system.

### Assays for proliferation and apoptosis

Sagittal sections obtained from the vermis region were exclusively used for each assay. To quantify proliferative GCPs, Phospho-Histone H3-positive cells were counted only in the EGL and normalized to the area of the EGL using Image J software (http://rsbweb.nih.gov/ij/). For quantification, data from at least three independent littermates were used. Apoptotic cells were labeled with anti-Cleaved Caspase-3 antibodies. Caspase-3 positive cells were examined in the EGL and in the entire cerebellum.

### Western blot analysis

The cerebellums of embryos were removed by dissection and were homogenized with lysis buffer containing 20 mM Tris-HCl (pH7.5), 150 mM NaCl, 1 mM Na_2_EDTA, 1 mM EGTA, 1% NP-40, 1% SDS, 2.5 mM sodium pyrophosphate, 1 mM Na_2_VO_4_, and Complete Protease Inhibitor Cocktail (Roche Applied Bioscience, Mannheim, Germany) per manufacturer's instructions. Protein extracts were resolved by SDS-PAGE and transferred to nitro cellulose membranes. These membranes were probed with anti-SHH (sc-9024, 1∶500; Santa Cruz Biotechnology, Santa Cruz, CA, USA) and anti-HSP70 (#4872, 1∶1000; Cell Signaling, Danvers, MA, USA) antibodies. Each blot was visualized using a SuperSignal West Pico Chemiluminescent detection kit (Thermo Scientific, Rockford, IL, USA) per manufacturer's instructions and quantified using ImageJ software (http://rsbweb.nih.gov/ij/). The expression level of SHH was normalized by the intensity of the HSP70 band in the same blot. Data from three cerebellums of each genotype were used for quantification.

## Results

### Expression of RERE in the developing mouse cerebellum

Although Zoltewicz et al. demonstrated that *Rere* is broadly expressed between E8.5 and E11.5—with expression being detected in the notochord, the ventral diencephalon, the spinal cord, and the optic vesicles—the expression of RERE has not been described in the developing cerebellum [24]. To evaluate the expression pattern of RERE, we performed immunohistochemical analyses on embryos and mice harvested at E15.5, E17.5, P0 and P14 using an antibody whose specificity was previously tested by western blot [25].

**Figure 1:**
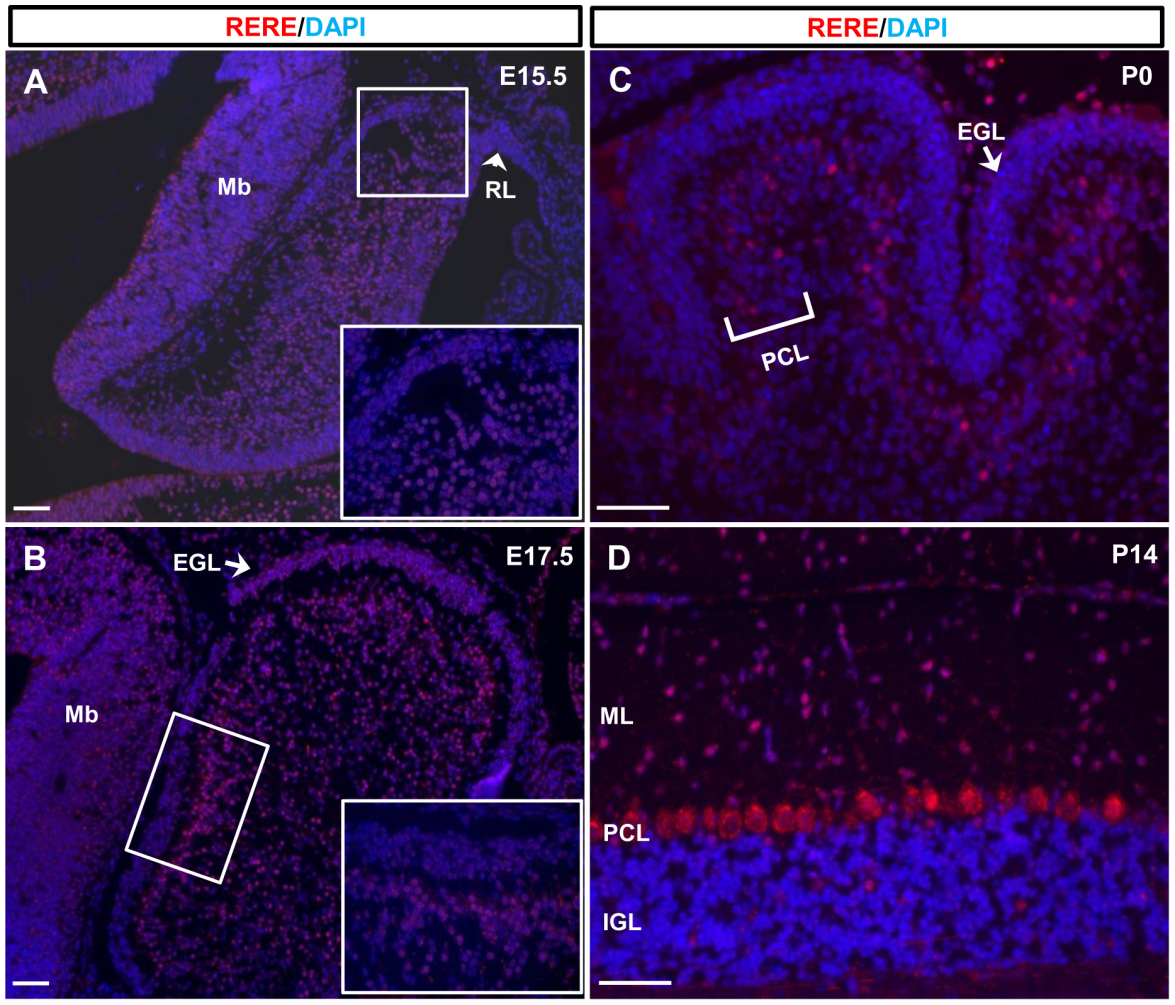
RERE is expressed in the developing cerebellum. A–D. Mid-sagittal sections of the vermis region were prepared from wild-type embryonic and postnatal mouse cerebellums at E15.5 (A), E17.5 (B), P0 (C), and P14 (D) and were stained with an anti-RERE antibody. RERE-positive cells were labeled with a red color and DAPI was used for counter staining. Inserts represent high magnification views of the boxed areas. A. RERE-positive cells are detected in the cortex of the cerebellum but not in the rhombic lip at E15.5. B. RERE expression is maintained in the entire cerebellum at E17.5. C–D. RERE immunoreactivity is particularly strong in the Purkinje cell layer at P0 (C) and P14 (D). EGL, external granule cell layer; IGL, internal granule cell layer; Mb, midbrain; ML, molecular layer; PCL, Purkinje cell layer; RL, rhombi lip. Scale bar  = 100 µm.

**Figure 2:**
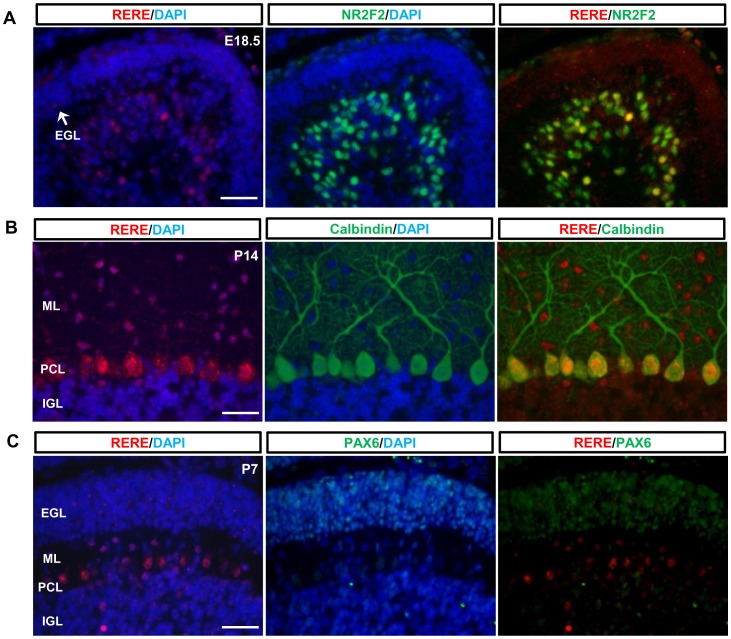
RERE is not expressed in granule cell precursors (GPCs) but is expressed in Purkinje cells. A–C. Mid-sagittal sections of the vermis region were prepared from wild-type mouse cerebellums at E18.5 (A), P14 (B) and P7 (C). A. Mid-sagittal sections of the vermis were stained with anti-RERE antibody (red color) and anti-NR2F2 antibody (green color). DAPI was used for counter staining. A subset of Purkinje cells labeled with NR2F2 is positive for RERE at E18.5. B. Purkinje cells labeled with mouse monoclonal anti-calbindin antibodies (green color) were also stained with anti-RERE antibodies at P14. RERE expressing cells were also identified in the molecular layer and in the internal granule cell layer at P14. C. RERE immunoreactivity was not detected in PAX6-positive cells in the vermis regions of wild-type cerebellums at P7. EGL, external granule cell layer; IGL, internal granule cell layer; ML, molecular layer; PCL, Purkinje cell layer. Scale bar  = 200 µm.

At E15.5, RERE-positive cells were broadly detected in the cerebellum but not in the rhombic lip where GCPs originate (Fig. 1A). At E17.5, RERE expression was still maintained in the entire cerebellum (Fig. 1B, Fig. S1A). Even though RERE-expressing cells were seen in the entire cerebellar region after birth, immunoreactivity of RERE was particularly strong in the PCL at P0, P7 and P14 (Figs. 1C and D, Fig. 2C, and Fig. S1A). At P14, RERE-positive cells were also located in the molecular layer (ML) and the internal granule cell layer (IGL) (Figs. 1D and 2B).

To determine if RERE was expressed in Purkinje cells at various times during cerebellar development, we used two different Purkinje cells markers: NR2F2, which is also known to be expressed in Purkinje cells during the early stages of cerebellar development [26], [27], [44] and calbindin which is a well-known marker for post-mitotic Purkinje cells [28]. At E18.5, a subset of Purkinje cells was positive for both RERE and NR2F2 antibodies but expression of RERE was not detected in all NR2F2-expressing cells (Fig. 2A). In contrast, all Purkinje cells were positive for both RERE and calbindin at P14 (Fig. 2B).

RERE immunoreactivity was seen in the EGL at E17.5 and P7 (Fig. 1B, Fig. 2C, and Fig. S1A) but was less intense than seen in other regions of the cerebellum, had a punctate pattern and did not always counterstain with DAPI. To determine if RERE was expressed in GCPs at these time points, we looked for co-expression of RERE and PAX6—a marker GCPs [29]. However, no RERE, PAX6 double positive cells were identified at E17.5 and P7 suggesting that RERE is not expressed in GCPs during development (Fig. 2C, Fig. S1B).

### Formation of the principal fissures is delayed in *Rere* ^om/eyes3^ embryos

**Figure 3:**
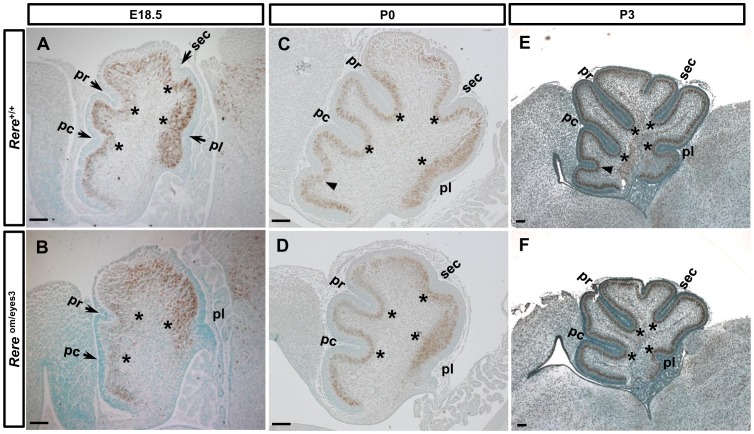
Foliation of the principal fissures is delayed in the cerebellums of *Rere* ^om/eyes3^ embryos. A–F. Mid-sagittal sections of the vermis were prepared from cerebellums of wild-type and *Rere* ^om/eyes3^ embryos and mice at E18.5 (A–B), P0 (B–C) and P3 (E–F). The Purkinje cell layer was marked using a rabbit polyclonal anti-calbindin antibody. Asterisks indicate the base of each principal fissure. A–B. At E18.5, all of the principal fissure were formed in the cerebellum of wild-type embryos (A) but the preculminate fissure, the primary fissure and the posterolateral fissure were barely appreciable in the cerebellums of *Rere* ^om/eyes3^ embryos (B). The secondary fissure was not seen in the cerebellums of *Rere* ^om/eyes3^ embryos (B). Arrows mark each principal fissure. C–D. Delayed development of principal fissures was restored in the cerebellums of *Rere* ^om/eyes3^ embryos at P0 (D). However, the precentral fissure was found in wild-type cerebellums (arrow head, C) but was not seen in the cerebellums of *Rere* ^om/eyes3^ mice (D) at P0. E–F. At P3, invagination of the precentral fissure (arrow head, E) demarcates the division of lobule III from lobule 1/II in the cerebellums of wild-type mice but the precentral fissure was not visible in the cerebellums of *Rere* ^om/eyes3^ mice (F). pc, preculminate fissure; pl, posterolateral fissure; pr, primary fissure; sec, secondary fissure. Scale bar  = 100 µm.

Since RERE is expressed in the cerebellum at E17.5 when foliation begins, we hypothesized that cerebellar foliation may be affected by RERE deficiency. To determine if this was true, we analyzed mid-sagittal sections of the cerebellar vermes of *Rere* ^om/eyes3^ and wild-type embryos and pups harvested at E18.5, P0 and P3. To visualize the anchoring of fissures, the PCL was stained using an anti-calbindin antibody. In wild-type embryos, the principal fissures were present at E18.5 with indentations of the EGL and PCL marking the locations of the preculminate, primary, secondary and posterolateral fissures (Fig. 3A). In the cerebellums from *Rere* ^om/eyes3^ embryos at the same time point, the secondary fissure was not visible (Fig. 3B). Invaginations corresponding to the preculminate, primary and posterolateral fissures were visible but their length was shorter than those of wild-type embryos (Fig. 3B).

At P0, the secondary fissure was formed in the cerebellums from *Rere* ^om/eyes3^ mice (Fig. 3D). Furthermore, development of principal fissures in *Rere* ^om/eyes3^ mice was comparable with the cerebellums of wild-type mice (Figs. 3C and D). However, the anchoring center of the precentral fissure was present in the cerebellums from wild-type mice but was absent in cerebellums from *Rere* ^om/eyes3^ mice (Figs. 3C and D). While indentation of the precentral fissure became deeper in the cerebellums of wild-type mice, the precentral fissure was still missing in the cerebellums of *Rere* ^om/eyes3^ mice at P3 (Figs. 3E and F). This suggests that the formation of the principal fissures and their corresponding anchoring centers is delayed in *Rere* ^om/eyes3^ embryos and the precentral fissure is not formed in *Rere* ^om/eyes3^ mice by P3.

### Reduction in proliferation of granule cell precursors and expression of SHH

Expansion of the number of GCPs is known to be a key driving force in cerebellar foliation [14]–[17], [30]. Mice lacking *Atoh1* (*Math1*) and *NeuroD1/β2* have foliation defects caused by loss of GCPs [30]–[32]. This led us to determine if the delayed foliation seen in *Rere* ^om/eyes3^ embryos and mice was accompanied by an increased level of GCP apoptosis. This was accomplished by staining for apoptotic cells in the EGL of *Rere* ^om/eyes3^ and wild-type embryos at E18.5 using an anti-Cleaved Caspase-3 antibody. However, no Cleaved Caspase-3-positive cells were detected in the EGLs of embryos of either genotype at E18.5 (Figs. S2A and B). In addition, apoptotic GCPs were not identified in the EGL of *Rere* ^om/eyes3^ mice and wild-type mice at P3 (Figs. S2C and D).

**Figure 4:**
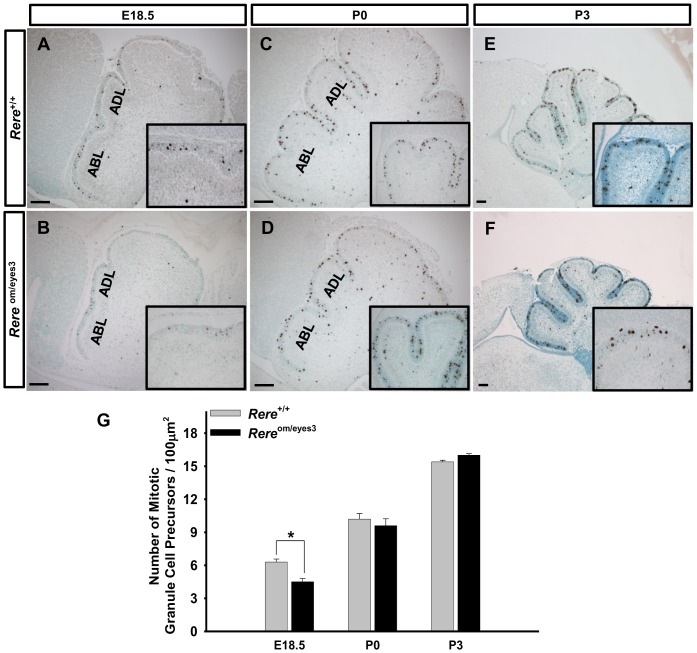
Granule cell precursor proliferation was decreased in the cerebellums of *Rere* ^om/eyes3^ embryos at E18.5. A-F. Proliferation assays were performed on cerebellar vermis sections prepared from the cerebellums of wild-type and *Rere* ^om/eyes3^ embryos and mice at E18.5 (A–B), P0 (C–D) and P3 (E–F) using an anti-Phospho Histone H3 antibody. A-B. The number of mitotic granule cell precursors in *Rere* ^om/eyes3^ embryos appeared to be lower than that of their wild-type littermates at E18.5. C-F. Mitotic activity of GCPs was comparable between *Rere* ^om/eyes3^ embryo and wild-type littermate at P0 and P3. G. Phospho-Histone H3-positive cells in the entire external granule cell layer (EGL) were counted and normalized by the area of the EGL (n≥3 with twenty slides containing three sections for each genotype). The number of Phospho-Histone H3-positive GCPs per µm^2^ was reduced in *Rere* ^om/eyes3^ embryos at E18.5 when compared to wild-type littermates (* = *p*<0.03) but was indistinguishable between *Rere* ^om/eyes3^ embryos and wild-type littermates at P0 and P3. ABL, anterobasal lobe; ADL, anterodorsal lobe. Scale bar  = 200 µm.

Next, we determined if the proliferative activity of the GCPs is affected in *Rere* ^om/eyes3^ embryos by labeling mitotic GCPs using Phospho-Histone H3 (pHH3) as a marker for cells that are in the mitotic phase (M phase) of the cell cycle. We found that the number of mitotic GCPs per µm^2^ was decreased in the EGLs of *Rere* ^om/eyes3^ embryos when compared with that of wild-type embryos at E18.5 (Figs. 4A, B and G). However, the number of mitotic GCPs per µm^2^ in the EGLs of *Rere* ^om/eyes3^ and wild-type mice at P0 and P3 was not statistically different (Figs. 4C–G).

The precentral fissure, in mice, is developed from the anterobasal lobe (ABL) around the time of birth. Since the precentral fissure is not identified in the cerebellums of *Rere* ^om/eyes3^ mice at P3, we questioned whether GCP proliferation in the ABL was reduced when compared to other areas of the cerebellum. To address this question, we compared the proliferative activity of GCPs in the ABL with the proliferative activity of GCPs in the adjacent anterodorsal lobe (ADL). Significant reductions in GCP proliferative activity were identified in both the ABLs and ADLs of *Rere* ^om/eyes3^ embryos at E18.5 when compared with those of their wild-type littermates (Fig. S3). However, the proliferative activity of GCPs was comparable between the ABL and ADL at this time point. At P0, the proliferative activity of GCPs was not affected in either the ABLs or the ADLs of *Rere* ^om/eyes3^ mice when compared with those of their wild-type littermates. The proliferative activity of GCPs was also comparable between the ABL and ADL at this time point (Fig. S3). This suggests that absence of the precentral fissure in *Rere* ^om/eyes3^ mice is not due to a specific reduction in GCP proliferation in the ABL.

**Figure 5:**
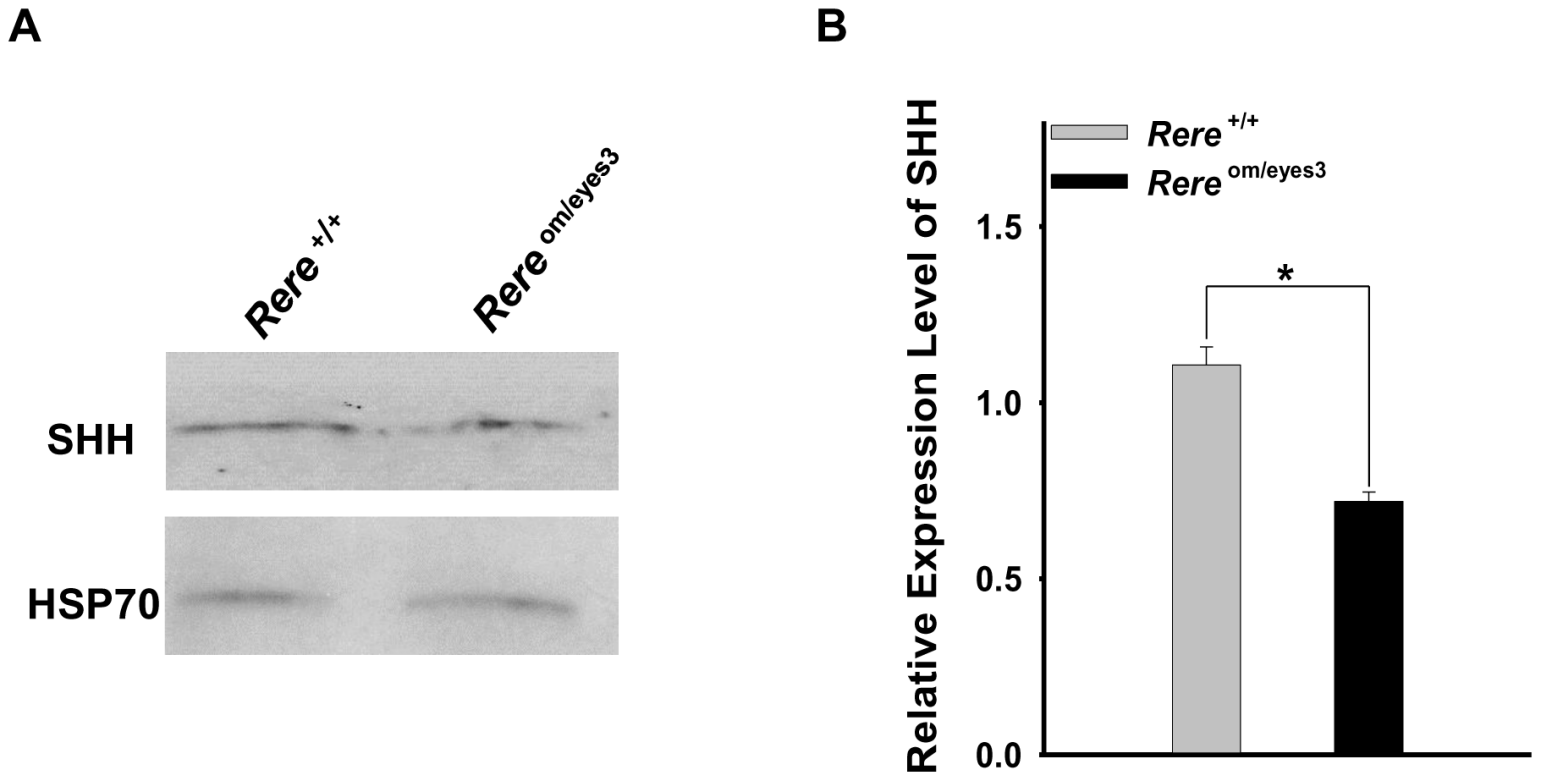
The level of SHH expression was reduced in the cerebellums of *Rere* ^om/eyes3^ embryos at E18.5. A. Protein extracts prepared from the cerebellums of *Rere* ^om/eyes3^ embryos and wild-type littermates at E18.5 were subjected to western blot analysis using an anti-SHH antibody and an anti-HSP70 antibody. Expression of SHH in the cerebellum of *Rere* ^om/eyes3^ embryos was lower than that of their wild-type littermates. Expression of HSP70 was comparable between *Rere* ^om/eyes3^ embryos and their wild-type littermates. B. To quantify the level of SHH expression, the density of the SHH band was normalized by the density of HSP 70 band in same blot. The expression level of SHH was significantly decreased in the cerebellums of *Rere* ^om/eyes3^ embryos when compared to the level of SHH in the cerebellums of their wild-type littermates at E18.5 (three independent experiments were performed for quantification; *  = *p*<0.01).

It has been shown that SHH is produced from Purkinje cells and acts as a mitogen to increase GCP proliferation in the mouse cerebellum [14], [15], [17], [19]–[20]. Since RERE was expressed in a subset of Purkinje cells at E18.5, and proliferation of GCPs was decreased in *Rere* ^om/eyes3^ embryos at that time point, we examined the expression level of SHH in the cerebellum of *Rere* ^om/eyes3^ embryos at E18.5 by western blot. We found that the expression of SHH was significantly lower in extracts from cerebellums obtained from *Rere* ^om/eyes3^ embryos when compared to extracts from cerebellums obtained from wild-type embryos (Figs. 5A and B).

### Migration and maturation of Purkinje cells is delayed in the developing cerebellums of *Rere* ^om/eyes3^ embryos

**Figure 6:**
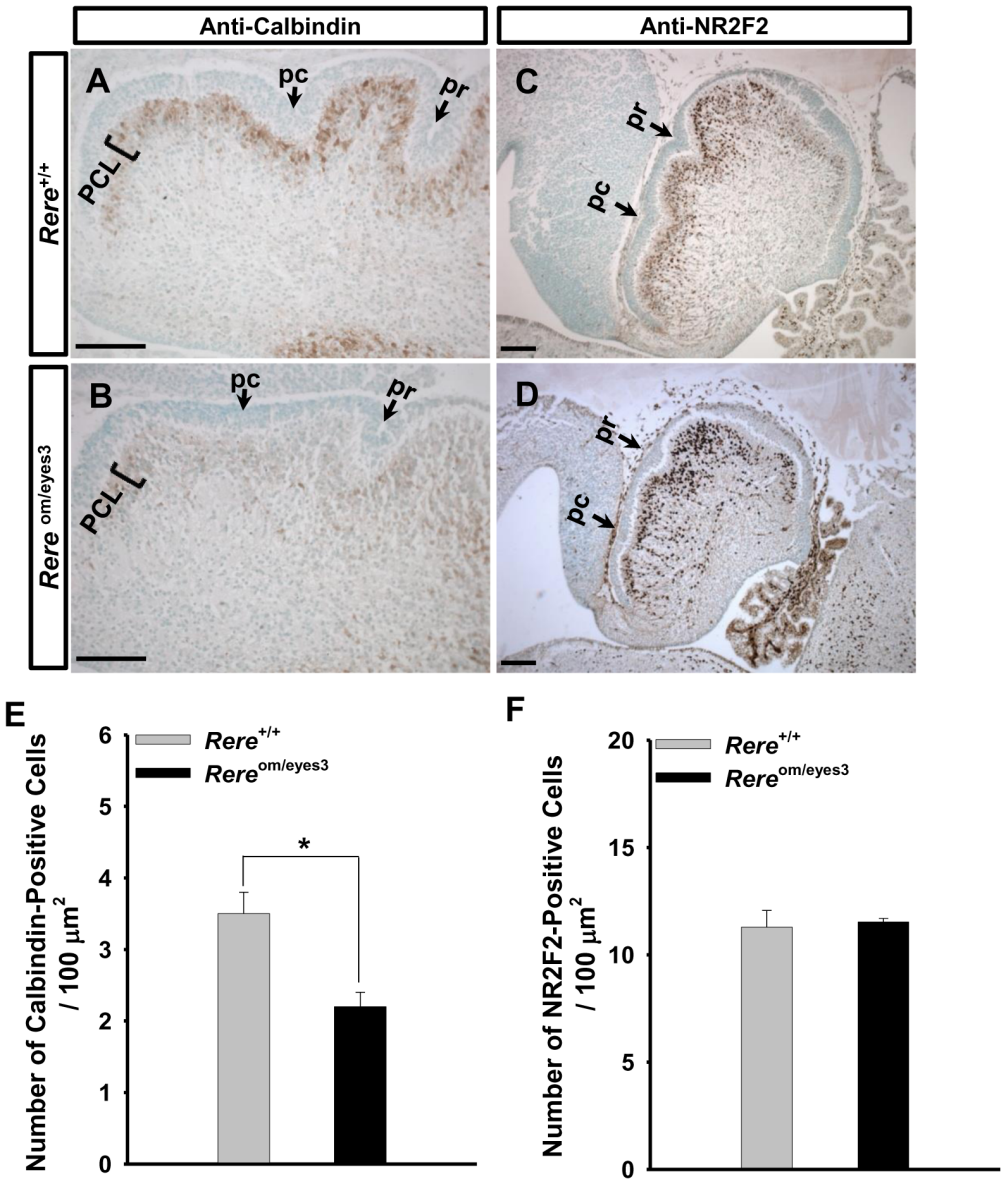
The migration and maturation of Purkinje cells was altered in *Rere* ^om/eyes3^ embryos at E18.5. A–D. Mid-sagittal sections of the vermis were probed with rabbit polyclonal anti-calbindin antibodies (A and B) or anti-NR2F2 antibodies (C and D). A–B. Number of calbindin-positive cells appeared to be lower in the cerebellums of *Rere* ^om/eyes3^ embryos (B) when compared with those of their wild-type littermates (A). C–D. In the cerebellums of wild-type embryos, most of the NR2F2-positive cells were located in the Purkinje cell layer underneath the external granule cell layer at E18.5 (C). In contrast, the number of NR2F2-positve cells was increased in the center of cerebellar cortex of *Rere* ^om/eyes3^ embryos at same time point (D). E-F. Calbindin-positive cells (E) and NR2F2-positive cells (F) were counted in the cerebellums of 18.5 embryos of both genotypes and normalized by the area of the cerebellum. The number of calbindin-positive cells/µm^2^ was significantly lower in the cerebellums of *Rere* ^om/eyes3^ embryos when compared with wild-type littermates (* = *p*<0.03). However, number of NR2F2-positive cells/µm^2^ was comparable between *Rere* ^om/eyes3^ embryos and their wild-type littermates. Quantification was performed using fifteen slides containing at least three sections from three independent littermates. pc, preculminate fissure; pr, primary fissure; PCL, Purkinje cell layer. Scale bar  = 100 µm.

In addition to a delay in foliation, we found that the number of calbindin-positive cells in the cerebellums of *Rere* ^om/eyes3^ embryos appeared to be less than that seen in wild-type littermates at E18.5 (Figs. 6A and B). To the quantify number of calbindin-positive cells, we counted calbindin-positive cells in the cerebellums of both genotypes and normalized by area. The number of calbindin-positive cells per µm^2^ was significantly decreased in the cerebellums of *Rere* ^om/eyes3^ embryos when compared with cerebellums obtained from their wild-type littermates at E18.5 (Fig. 6 E). Since calbindin is a marker of post-mitotic Purkinje cells, this suggested that the number of these cells is reduced in the cerebellums of *Rere* ^om/eyes3^ embryos.

To determine why the number of post-mitotic Purkinje cells was less in the *Rere* ^om/eyes3^ cerebellum, we first looked for evidence of increased Purkinje cell apoptosis by marking apoptotic cells using an anti-Cleaved Caspase-3 antibody. However, no Caspase-3-positive cells were detectable in the PCL of *Rere* ^om/eyes3^ embryos or their wild-type embryo littermates, suggesting that an increase in Purkinje cell apoptosis was not the cause of reduced post-mitotic Purkinje cell counts at E18.5 (Figs. S2A and B).

Since apoptosis was not associated with reduced number of Purkinje cells in the cerebellums of *Rere* ^om/eyes3^ embryos, we looked for evidence that Purkinje cells are not properly recruited in the cerebellums of *Rere* ^om/eyes3^ embryos. To do so, we used NR2F2 as a marker for early Purkinje cells since it is known to be expressed in the cerebellar analge as early as E13.0 and in migrating Purkinje cells starting at E15.5 [27]. In the cerebellums of wild-type embryos at E18.5, most NR2F2-positive cells were located in the PCL and only a few NR2F2-positive cells were identified in the center of cerebellar cortex (Fig. 6C). This pattern is similar to the distribution of calbindin-positive cells in wild-type embryos at this time point (Fig. 6A). In comparison to their wild-type littermates, *Rere* ^om/eyes3^ embryos had more NR2F2-positive cells located in the center of cerebellar cortex at E18.5 (Figs. 6C and D) and the distribution pattern of NR2F2-positive cells was wider than that of calbindin-positive cells in *Rere* ^om/eyes3^ embryos at the same time point (Fig. 6B and D).

Although the number of calbindin positive cells per µm^2^ of cerebellar cortex was decreased in *Rere* ^om/eyes3^ embryos compared to their wild-type littermates, we found that the number of NR2F2-positive cells per µm^2^ of cerebellar cortex was comparable between embryos of both genotypes at E18.5 (Fig. 6F).

Taken together, our results suggest that although the total number of Purkinje cells is comparable between *Rere* ^om/eyes3^ and wild-type embryos, the migration and maturation of Purkinje cells in the cerebellums of *Rere* ^om/eyes3^ embryos is delayed.

### RERE deficiency results in abnormal foliation in the cerebellums of *Rere* ^om/eyes3^ mice

**Figure 7:**
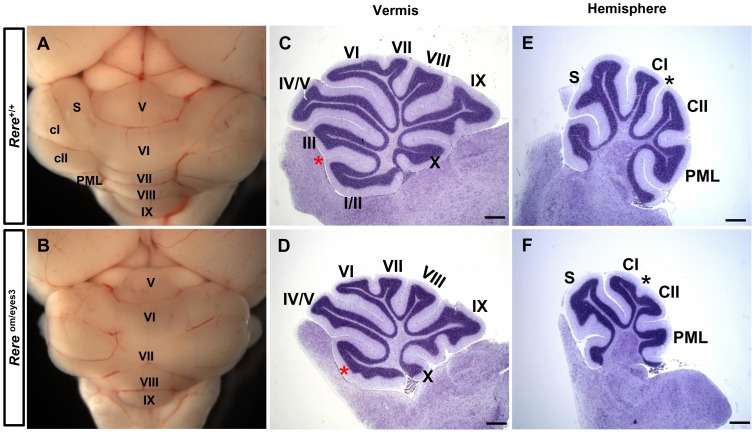
*Rere* ^om/eyes3^ mice have cerebellar foliation defects at P21. A–B. The cerebellar vermis and hemispheres were evident in wild-type and *Rere* ^om/eyes3^ mice. C–D. Mid-sagittal sections of the vermis region were stained with cresyl violet. In the cerebellums of *Rere* ^om/eyes3^ mice, the precentral fissure was severely attenuated and failed to divide lobule I/II from lobule III. Red asterisks indicate the location of the precentral fissure in wild-type mice (C) and marks the abnormal lobule in *Rere* ^om/eyes3^ mice (D). E–F. Sagittal sections of the cerebellar hemispheres were stained with cresyl violet. The intercrural fissure was fully developed in wild-type mice (E), but was severely attenuated in the cerebellums of *Rere* ^om/eyes3^ mice (F). The black asterisks indicate the intercrural fissure. Roman letters mark corresponding lobules in the vermis of the cerebellum. CI, lobule crus I; CII, lobule crus II; PML, paramedian lobe; S, simple lobule. Scale bar  = 500 µm.

The majority of *Rere* ^om/eyes3^ mice die in the perinatal period with cardiac and renal defects [25]. However, a subset survives into adulthood allowing us to determine the effects of RERE-depletion on adult cerebellar morphology. Along the medial-lateral axis, the external morphological land marks of the cerebellum—including the vermis and hemispheres—were identified in both *Rere* ^om/eyes3^ mice and wild-type mice after gross dissection at P21 (Figs. 7A and B). However the cerebellar vermes and cerebellar hemispheres of *Rere* ^om/eyes3^ mice were smaller than those of their wild-type littermates (Figs. 7A and B). These size differences may be due, in part, to the postnatal growth retardation that has been previously documented in *Rere* ^om/eyes3^ mice [25].

In sagittal sections prepared from the vermes of wild-type mice, the anterobasal lobe was completely divided into lobule I/II and lobule III by the precentral fissure at P21 (Fig 7C). In contrast, lobule I/II and lobule III failed to separate at P21 in *Rere* ^om/eyes3^ mice due to severe attenuation of the precentral fissure (Fig. 7D).

Abnormalities in cerebellar foliation were also evident in the cerebellar hemispheres of *Rere* ^om/eyes3^ mice. In sagittal sections of the cerebellar hemispheres of wild-type mice, lobule crus I and lobule crus II were fully divided by the intercrural fissure at P21 (Fig. 7E). In *Rere* ^om/eyes3^ mice, development of the intercrural fissure was severely attenuated and failed to separate lobule crus I from lobule crus II at P21 (Fig. 7F).

### Decreased branching of Purkinje cell dendrites in the cerebellums of *Rere* ^om/eyes3^ mice

**Figure 8:**
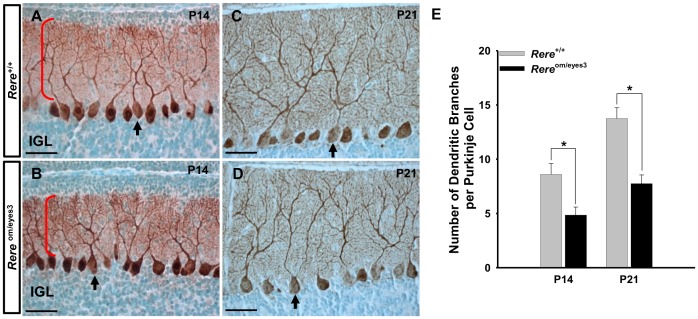
Branching of Purkinje cell dendrites was reduced in the cerebellums of *Rere* ^om/eyes3^ mice. A–D. Sagittal sections of the vermis of *Rere* ^om/eyes3^ mice and their wild-type littermates were probed with rabbit polyclonal anti-calbindin antibodies at P14 (A, B) and P21 (C, D). A–D. A monolayer of Purkinje cells was found in the cerebellums of mice of both genotypes. Arrows point to individual Purkinje cells. Red brackets mark the dendrites of the Purkinje cells at P14. The Purkinje cell dendrites branching of *Rere* ^om/eyes3^ mice (B, D) appeared to be decreased when compared that of their wild-type littermates (A, C). E. Quantification of Purkinje cells branches revealed that the Purkinje cells of *Rere* ^om/eyes3^ mice at both P14 and P21 have significantly fewer branches compared to the Purkinje cells of wild-type mice at the same time points (* = *p*<0.03). Purkinje cells containing at least two secondary dendrites were exclusively used for quantification of dendritic branching. Quantification was performed using ten slides containing at least three sections from three independent littermates. IGL, internal granule cell layer. Scale bar  = 50 µm.

Since RERE is expressed in Purkinje cells during the postnatal period (Figs. 1D and 2B–C), we questioned whether postnatal Purkinje cells maturation was affected by RERE deficiency. Cerebellar sections from *Rere* ^om/eyes3^ mice and wild-type littermates at P14 and P21 were stained with an anti-calbindin antibody. We found a monolayer of Purkinje cells in cerebellums harvested from mice of both genotypes at P14 and P21 (Figs. 8A–D). However, the Purkinje cell dendrites of the *Rere* ^om/eyes3^ mice appeared to have decreased arborization when compared to those of wild-type mice (Figs. 8A vs. B and 8C vs. D). When the number of high order dendritic branches was counted, we found that the average number of Purkinje cell dendrites in *Rere* ^om/eyes3^ mice was significantly decreased when compared to their wild-type littermates at P14 and P21 (Fig. 8E).

## Discussion

### RERE is required for normal prenatal and postnatal cerebellar development

A number of nuclear receptors and their associated coregulators have been shown to play key roles in the temporospatial regulation of gene expression which is required for proper development of the central nervous system. RERE is a nuclear receptor coregulator that is widely expressed in the developing central nervous system and is required for normal brain development. Zoltewicz et al. demonstrated that a complete lack of RERE causes severe defects in CNS development with *Rere* ^om/om^ embryos displaying fusion of the telencephalic vesicles, defects of the optic vesicles, and failure of the anterior neural tube closure at E9.5 [24]. Subsequently, we used RERE-deficient *Rere* ^om/eyes3^ embryos to shown that RERE deficiency causes brain hypoplasia and decreased numbers of neuronal nuclear antigen (NeuN)-positive hippocampal neurons [25].

To determine if RERE plays a specific role in cerebellar development, we first determined the expression pattern of RERE in the cerebellum at various time points in its development. Zoltewicz et al. had previously shown that RERE is expressed in the isthmus [24], a signaling center at the junction of the mesencephalon and the metencephalon which plays a critical role in regulating cerebellar development [33]–[36]. In this study, we determined that RERE was broadly expressed in the cortex of the cerebellar primordium from E15.5 to E17.5. We also found that RERE was expressed in the postnatal cerebellum with particularly strong expression in Purkinje cells at P0, P7 and P14.

Even though RERE-positive cells were identified in the internal granule cell layer, these cells did not colocalize with PAX6 immunostaining at P7. This suggests that RERE is not expressed in the granule cells of the IGL but may, instead, be expressed in Golgi cells, Lugaro cells and/or the unipolar brush cells at this time point.

Based on these findings, we performed a detailed analysis of cerebellar development using *Rere* ^om/eyes3^ embryos and mice. This analysis revealed that RERE plays a role in both prenatal and postnatal cerebellar development and that RERE depletion leads to abnormal cerebellar foliation, decreased GCP proliferation associated with altered levels of SHH, delay of Purkinje cell maturation and migration, and reduced branching of Purkinje cell dendrites. These findings are discussed in greater detail in the subsections that follow.

### Abnormal foliation of the cerebellums of *Rere* ^om/eyes3^ mice

Although the principal fissures and cardinal lobes were seen in the cerebellums of *Rere* ^om/eyes3^ mice after birth, we discovered that RERE deficiency causes a delay in the foliation process during the prenatal period. In wild-type cerebellums, invagination of the EGL and the PCL mark the anchoring centers of the principal fissures at E18.5. These areas will ultimately form the bases of the principal fissures. In contrast, invagination of the EGL and the PCL was limited in the cerebellums of *Rere* ^om/eyes3^ embryos at E18.5 and the secondary fissure was not anchored. Furthermore, the principal fissures of E18.5 *Rere* ^om/eyes3^ embryos were shorter than the principal fissures of wild-type embryos at the same time point. At P0 and P3, all of the principal fissures, including the secondary fissure, were identified in the cerebellums of *Rere* ^om/eyes3^ mice.

Foliation of non-principal fissures begins after birth and undergoes maturation during postnatal development in the mouse cerebellum [1], [12]. In wild-type mice, the anterobasal lobe undergoes further foliation by invagination of the precentral fissure at P0 and lobule III is fully separated from lobule I/II by the precentral fissure at P3. In contrast, in *Rere* ^om/eyes3^ mice, there was no evidence of precentral fissure anchoring at P3 and lobule I/II and lobule III failed to separate at P21 due to severe attenuation of the precentral fissure.

In the cerebral hemispheres of wild-type mice, the intercrural fissure divides the ansiform lobule into lobule Crus I and lobule Crus II. In *Rere* ^om/eyes3^ mice, development of the intercrural fissure was limited with failure of the ansiform lobule to subdivide into lobule crus I and lobule crus II at P21.

Based on these data we conclude that RERE deficiency causes a delay in principal fissure initiation during prenatal cerebellar development and that RERE is required for normal postnatal foliation of the cerebellar vermis and hemispheres.

### Decreased proliferation of GCPs and expression of SHH in *Rere* ^om/eyes3^ embryo cerebellum

Expansion in the number of GCP is known to be a driving force in the foliation of the cerebellum [14], [16]–[18], [30]. Our studies did not find evidence of increased GCP apoptosis in *Rere* ^om/eyes3^ embryos. Instead, we found evidence that RERE deficiency causes a decrease in the proliferative activity of GCPs in the cerebellums of *Rere* ^om/eyes3^ embryos at E18.5 when compared to wild-type littermates. In contrast, at P0 and P3, GCP proliferation was comparable between *Rere* ^om/eyes3^ mice and their wild-type littermates. From E18.5 to P3, GCP proliferation was comparable between the anterobasal lobe and the anterodorsal lobe, suggesting that absence of the precentral fissure in *Rere* ^om/eyes3^ mice is not due to a specific reduction in GCP proliferation in the anterobasal lobe. Since RERE expression was not detected in the rhombic lip at E15.5 or in PAX6-positive GCPs at E18.5, we conclude that RERE deficiency affects GCP proliferation prior to birth in non-cell-autonomous manner.

In mice, Purkinje cell depletion has been shown to cause defects in foliation associated with reduced rates of GCPs proliferation, emphasizing the importance of cross talk between these cell types during cerebellar development [37], [38]. It is well established that SHH is expressed in Purkinje cells and that the SHH signaling pathway is critical for GCP proliferation [16], [18]–[20]. Since RERE is expressed in a subset of Purkinje cells at E18.5, and the mitotic activity of GCPs was decreased in *Rere* ^om/eyes3^ embryos at the same time, we hypothesized that RERE could be regulating GCP proliferation by modulating the expression of SHH. When we tested this hypothesis, we found that expression of SHH was significantly decreased at E18.5 in the cerebellums of *Rere* ^om/eyes3^ embryos.

Taken together, these results suggest that RERE deficiency contributes to delay of principal fissure formation through decreased proliferation of GCPs which, in turn, is caused by reduced expression of SHH in the prenatal cerebellum. Whether this reduction in prenatal SHH expression is due to RERE's cell autonomous effects in Purkinje cells remains to be determined.

### Purkinje cell abnormalities in *Rere* ^om/eyes3^ embryos

In mice, Purkinje cell precursors migrate out along the radial-glial-fiber system after E13 and settle in the cortex area of the cerebellar primordium forming a thick and dense zone of Purkinje cells known as the Purkinje cell plate around E15.5 [39], [40]. The Purkinje cells within the Purkinje cell plate form multiple layers along the principal fissures at E18.5, which are maintained until a few days after birth [39], [40]. *Rere* ^om/eyes3^ embryos at E18.5 had decreased numbers of post-mitotic Purkinje cells per area in the cerebellum when compared to their wild-type littermates. At the same time point, the number of NR2F2-positive Purkinje cells was comparable between *Rere* ^om/eyes3^ embryos and their littermates but a greater number of migrating NR2F2-positive Purkinje cells were detected in *Rere* ^om/eyes3^ embryos. These data suggest that migration of Purkinje cells is delayed in *Rere* ^om/eyes3^ embryos. In addition, the delayed maturation of Purkinje cells in *Rere* ^om/eyes3^ embryos—evidenced by a decreased number of calbindin-positive Purkinje cells/per area compared to wild-type mice at E18.5—may be responsible for the reduced expression of SHH detected in *Rere* ^om/eyes3^ embryos at the same time point.

Since RERE is expressed in Purkinje cells at P14, we hypothesized that RERE may also have effects on postnatal Purkinje cell maturation. When we examined cerebellums at P14 and P21, we found that Purkinje cells were organized in a monolayer in both wild-type and *Rere* ^om/eyes3^ mice. However, closer examination revealed that the level of Purkinje cell dendrite branching was significantly decreased in *Rere* ^om/eyes3^ mice compared to their wild-type littermate controls at these time points. This suggests that RERE depletion also leads to abnormal postnatal maturation of Purkinje cells.

It is possible that RERE's effects on Purkinje cells migration and maturation are due to its cell-autonomous role within these cells. Testing this hypothesis, and dissecting out RERE's role in other cell types within the cerebellum, will require the use of *Rere* conditional knockout mice. These mice will also be useful in determining if RERE depletion in the cerebellum leads to abnormalities in motor coordination—something which cannot be easily determined in *Rere* ^om/eyes3^ mice due to the potential confounding effects of systemic RERE depletion [25].

### Nuclear receptors in cerebellar development

RERE's role as a nuclear receptor coregulator suggests that it functions during cerebellar development to mediate the effects of one or more nuclear receptors. RERE has been shown to interact with members of nuclear receptor subfamily 2 (NR2) such as NR2E1 and NR2F2 [22], [23]. NR2E1-deficient mice can have hypoplasia of rhinencephalic and limbic structures—including the olfactory, infra-rhinal and entorhinal cortex, amygdala and dentate gyrus—enlarged ventricles, hydrocephalus, retinal dystrophy, blindness and aggression. However, cerebellar defects have not been described as a consequence of decreased NR2E1 expression, even though *NR2E1* has been shown to be expressed in the human cerebellum [41]–[43].

NR2F2 has been documented to play a critical role in cerebellum development, but the expression pattern of NR2F2 in the cerebellum, and many of its effects on cerebellar development, are distinct from those of RERE [44]. Specifically, NR2F2 is expressed exclusively in the Purkinje cells of the cerebellum. In the prenatal stage, delay of foliation was found in *Rere* ^om/eyes3^ embryos but was not seen in embryos in which *Nr2f2* expression was conditionally ablated in neurons using a transgenic Nse-Cre (*Nr2f2* ^flox/flox^;Nse-Cre) [44]. Postnatally, foliation defects are seen in both *Rere* ^om/eyes3^ and *Nr2f2* ^flox/flox^; Nse-Cre mice but truncated development of the precentral fissure leads to incomplete division of lobules I/II and III in RERE-deficient mice while NR2F2 depletion led to failure of lobules VIa and VIb to be properly divided. This makes it unlikely that RERE and NR2F2 participate together in regulating these aspects of cerebellar development.

Depletion of both RERE and NR2F2 have a detrimental effect on Purkinje cell maturation with the Purkinje cells of both *Rere* ^om/eyes3^ and *Nr2f2* ^flox/flox^;Nse-Cre mice having decreased dendrite branching. Future studies will be needed to determine if RERE interacts with NR2F2 to affect Purkinje cell maturations and to identify the other nuclear receptors that interact with RERE during cerebellar development.

## Supporting Information

Figure S1 **RERE is expressed in the developing cerebellum but not in granule cell precursors.** A–B. Mid-sagittal sections of the vermis region were prepared from wild-type embryonic and postnatal mouse cerebellums at E17.5 (A, B) and P7 (A) and were stained with an anti-RERE antibody. RERE-positive cells were labeled with a red color (A and B; Alexa 568) and PAX6-positive cells were visualized with a green color (B; Alexa 488). A. Immunoreactivity for anti-RERE antibodies is detected in the external granule cell layer (EGL) and other areas of the cerebellum at E17.5 and P7. B. RERE, PAX6 double positive cells are not identified in the cerebellum at E17.5 suggesting that RERE is not expressed in granule cell precursors. Scale bar  = 100 µm. IGL, internal granule cell layer.
(DOCX)

Figure S2 **Apoptotic activity in the cerebellum was comparable between** ***Rere*** **^om/eyes3^ embryos and controls.** A–D Mid-sagittal sections from embryos and mice of both genotypes were probed with anti-Cleaved Caspase-3 antibodies to label apoptotic cells at E18.5 (A, B) and P3 (C, D). A-B. A few Cleaved Caspase-3-positive cells were identified in the cerebellar cortexes of *Rere* ^om/eyes3^ embryos and their wild-type littermates at E18.5. In contrast, apoptotic cells were undetectable in the EGLs and PCLs of *Rere* ^om/eyes3^ embryos and their wild-type littermates. C–D. Apoptotic activity of the cerebellums between *Rere* ^om/eyes3^ mice and their wild-type littermates was comparable at P3. Scale bar  = 100 µm. EGL, external granule cell layer; Mb, midbrain; PCL, Purkinje cell layer.
(DOCX)

Figure S3 **Mitotic activity of granule cell precursors was comparable between the anterobasal and anterodorsal lobes.** At E18.5 and P0, proliferation assays were performed on cerebellar vermis sections prepared from wild-type and *Rere* ^om/eyes3^ embryos and mice using an anti-Phospho Histone H3 antibody. The number of Phospho-Histone H3-positive granule cell precursors (GCPs) in the anterobasal lobe (ABL) and the anterodorsal lobe (ADL) was reduced in *Rere* ^om/eyes3^ embryos at E18.5 when compared to wild-type littermates (* = *p*<0.03). However, the proliferative activity of GCPs was comparable between the ABLs and ADLs of *Rere* ^om/eyes3^ embryos at same time point. At P0, the mitotic activity of GCPs in the ABL and ADL was indistinguishable between *Rere* ^om/eyes3^ embryos and wild-type littermates. In addition, proliferative activity of GCPs was similar between the ABLs and ADLs of *Rere* ^om/eyes3^ mice at P0. Phospho-Histone H3-positive cells in the external granule cell layer (EGL) of the ABL and ADL were counted and normalized by the corresponding area of the EGL of each lobe (n≥3 with twenty slides containing three sections for each genotype).
(DOCX)

Table S1 **Additional information about the antibodies used in this study.**
(DOCX)
