# Supplementary material for: Mouse Model Reveals the Role of RERE in Cerebellar Foliation and the Migration and Maturation of Purkinje Cells
Source: PLoS One. 2014 Jan 23;9(1):e87518. doi: 10.1371/journal.pone.0087518 (PMC3900724; doi:10.1371/journal.pone.0087518)
Supplement: Figure S1 — RERE is expressed in the developing cerebellum but not in granule cell precursors. A–B. Mid-sagittal sections of the vermis region were prepared from wild-type embryonic and postnatal mouse cerebellums at E17.5 (A, B) and P7 (A) and were stained with an anti-RERE antibody. RERE-positive cells were labeled with a red color (A and B; Alexa 568) and PAX6-positive cells were visualized with a green color (B; Alexa 488). A. Immunoreactivity for anti-RERE antibodies is detected in the external granule cell layer (EGL) and other areas of the cerebellum at E17.5 and P7. B. RERE, PAX6 double positive cells are not identified in the cerebellum at E17.5 suggesting that RERE is not expressed in granule cell precursors. Scale bar = 100 µm. IGL, internal granule cell layer. (DOCX) [file pone.0087518.s001.docx]

**Figure S1. RERE is expressed in the developing cerebellum but not in granule cell precursors.** A-B. Mid-sagittal sections of the vermis region were prepared from wild-type embryonic and postnatal mouse cerebellums at E17.5 (A, B) and P7 (A) and were stained with an anti-RERE antibody. RERE-positive cells were labeled with a red color (A and B; Alexa 568) and PAX6-positive cells were visualized with a green color (B; Alexa 488). A. Immunoreactivity for anti-RERE antibodies is detected in the external granule cell layer (EGL) and other areas of the cerebellum at E17.5 and P7. B. RERE, PAX6 double positive cells are not identified in the cerebellum at E17.5 suggesting that RERE is not expressed in granule cell precursors. Scale bar = 100 µm. IGL, internal granule cell layer.
