# Supplementary material for: Mouse Model Reveals the Role of RERE in Cerebellar Foliation and the Migration and Maturation of Purkinje Cells
Source: PLoS One. 2014 Jan 23;9(1):e87518. doi: 10.1371/journal.pone.0087518 (PMC3900724; doi:10.1371/journal.pone.0087518)
Supplement: Figure S2 — Apoptotic activity in the cerebellum was comparable between Rere om/eyes3 embryos and controls. A–D Mid-sagittal sections from embryos and mice of both genotypes were probed with anti-Cleaved Caspase-3 antibodies to label apoptotic cells at E18.5 (A, B) and P3 (C, D). A-B. A few Cleaved Caspase-3-positive cells were identified in the cerebellar cortexes of Rere om/eyes3 embryos and their wild-type littermates at E18.5. In contrast, apoptotic cells were undetectable in the EGLs and PCLs of Rere om/eyes3 embryos and their wild-type littermates. C–D. Apoptotic activity of the cerebellums between Rere om/eyes3 mice and their wild-type littermates was comparable at P3. Scale bar = 100 µm. EGL, external granule cell layer; Mb, midbrain; PCL, Purkinje cell layer. (DOCX) [file pone.0087518.s002.docx]

**Figure S2**. **Apoptotic activity in the cerebellum was comparable between *Rere*^om/eyes3^ embryos and controls.** A-D Mid-sagittal sections from embryos and mice of both genotypes were probed with anti-Cleaved Caspase-3 antibodies to label apoptotic cells at E18.5 (A, B) and P3 (C, D). A-B. A few Cleaved Caspase-3-positive cells were identified in the cerebellar cortexes of *Rere*^om/eyes3^ embryos and their wild-type littermates at E18.5. In contrast, apoptotic cells were undetectable in the EGLs and PCLs of *Rere*^om/eyes3^ embryos and their wild-type littermates. C-D. Apoptotic activity of the cerebellums between *Rere*^om/eyes3^ mice and their wild-type littermates was comparable at P3. Scale bar = 100 µm. EGL, external granule cell layer; Mb, midbrain; PCL, Purkinje cell layer.
