# Supplementary material for: Mouse Model Reveals the Role of RERE in Cerebellar Foliation and the Migration and Maturation of Purkinje Cells
Source: PLoS One. 2014 Jan 23;9(1):e87518. doi: 10.1371/journal.pone.0087518 (PMC3900724; doi:10.1371/journal.pone.0087518)
Supplement: Figure S3 — Mitotic activity of granule cell precursors was comparable between the anterobasal and anterodorsal lobes. At E18.5 and P0, proliferation assays were performed on cerebellar vermis sections prepared from wild-type and Rere om/eyes3 embryos and mice using an anti-Phospho Histone H3 antibody. The number of Phospho-Histone H3-positive granule cell precursors (GCPs) in the anterobasal lobe (ABL) and the anterodorsal lobe (ADL) was reduced in Rere om/eyes3 embryos at E18.5 when compared to wild-type littermates (* = p<0.03). However, the proliferative activity of GCPs was comparable between the ABLs and ADLs of Rere om/eyes3 embryos at same time point. At P0, the mitotic activity of GCPs in the ABL and ADL was indistinguishable between Rere om/eyes3 embryos and wild-type littermates. In addition, proliferative activity of GCPs was similar between the ABLs and ADLs of Rere om/eyes3 mice at P0. Phospho-Histone H3-positive cells in the external granule cell layer (EGL) of the ABL and ADL were counted and normalized by the corresponding area of the EGL of each lobe (n≥3 with twenty slides containing three sections for each genotype). (DOCX) [file pone.0087518.s003.docx]

**Figure S3**. **Mitotic activity of granule cell precursors was comparable between the anterobasal and anterodorsal lobes.** At E18.5 and P0, proliferation assays were performed on cerebellar vermis sections prepared from wild-type and *Rere*^om/eyes3^ embryos and mice using an anti-Phospho Histone H3 antibody. The number of Phospho-Histone H3-positive granule cell precursors (GCPs) in the anterobasal lobe (ABL) and the anterodorsal lobe (ADL) was reduced in *Rere*^om/eyes3^ embryos at E18.5 when compared to wild-type littermates (* = *p <* 0.03). However, the proliferative activity of GCPs was comparable between the ABLs and ADLs of *Rere*^om/eyes3^ embryos at same time point. At P0, the mitotic activity of GCPs in the ABL and ADL was indistinguishable between *Rere*^om/eyes3^ embryos and wild-type littermates. In addition, proliferative activity of GCPs was similar between the ABLs and ADLs of *Rere*^om/eyes3^ mice at P0. Phospho-Histone H3-positive cells in the external granule cell layer (EGL) of the ABL and ADL were counted and normalized by the corresponding area of the EGL of each lobe (n ≥ 3 with twenty slides containing three sections for each genotype).
