# Supplementary material for: Mouse Model Reveals the Role of RERE in Cerebellar Foliation and the Migration and Maturation of Purkinje Cells
Source: PLoS One. 2014 Jan 23;9(1):e87518. doi: 10.1371/journal.pone.0087518 (PMC3900724; doi:10.1371/journal.pone.0087518)
Supplement: Table S1 — Additional information about the antibodies used in this study. (DOCX) [file pone.0087518.s004.docx]

**Table S1. Additional information about the antibodies used in this study.**

| **Primary Antibody (Catalog number, Supplier)** | **Host** | **Specificity/Application/References** |
| --- | --- | --- |
| Anti-Calbindin (AB1778, Millipore) | Rabbit | This antibody does not cross react with calretinin by WB. WB data was presented by the manufacturer ([http://www.millipore.com/ catalogue/item/ab1778#](http://www.millipore.com/%20catalogue/item/ab1778%23)). Use for immunohistochemistry IHC was previously reported [1]. |
| Anti-Calbindin (C9848, Sigma) | Mouse | This antibody does not react with other members of the EF-hand family such as calbindin-D-9K, calretinin, myosin light chain, parvalbumin, S-100a, S-100b, S-100A2 (S100L) and S-100A6 (calcyclin) ([http://www.sigmaaldrich.com/content/dam/sigma-aldrich/docs /Sigma/Datasheet/3/c9848dat.pdf](http://www.sigmaaldrich.com/content/dam/sigma-aldrich/docs%20/Sigma/Datasheet/3/c9848dat.pdf)). Use for IHC was previously reported [2]. |
| Anti-Cleaved Caspase-3 (#9664, Cell Signaling) | Rabbit | This antibody detects the large fragment (17/19 kDa) of activated caspase-3 resulting from cleavage adjacent to Asp175. This antibody does not recognize full length caspase-3 or other cleaved caspases (<http://www.cellsignal.com/pdf/9664.pdf>). Use for WB and IHC was previously reported [3,4]. |
| Anti-HSP70 (#4872, Cell Signaling) | Rabbit | This antibody detects total HSP70 protein (<http://www.cellsignal.com/pdf/4872.pdf>). Use for WB was previously reported [5]. |
| Anti-NR2F2 (ab41859, Abcam) | Mouse | This antibody does not recognize human NR2F1 and NR2F6 (<http://www.abcam.com/nr2f2-antibody-h7147-ab41859.html>). Use for WB and IHC was previously reported [6,7]. |
| Anti-PAX6 (PAX6, Developmental Studies Hybridoma Bank) | Mouse | Details about this antibody are available at <http://dshb.biology.uiowa.edu/PAX6>. Use for IHC was previously reported [8]. |
| Anti-Phosho-Histone H3 (#9701, Cell Signaling) | Rabbit | This antibody detects histone H3 only when phosphorylated at serine 10. This antibody does not cross-react with other phosphorylated histones or with acetylated histones (<http://www.cellsignal.com/pdf/9701.pdf>). Use for WB and IHC was previously reported [9]. |
| Anti-RERE (sc-98415, Santa Cruz Biotech.) | Rabbit | Details about this antibody are available at <http://datasheets.scbt.com/sc-98415.pdf>. Use for WB and IHC was previously reported [10]. Using this antibody, we found no discernible RERE protein in *Rere*^om/om^ embryos at E10.5 by WB [10]. |
| Anti-SHH (sc-9024, Santa Cruz Biotech.) | Rabbit | Details about this antibody are available at <http://datasheets.scbt.com/sc-9024.pdf>. Use for WB and IHC was previously reported [11,12]. |

IHC = immunohistochemistry; WB = western blot

**REFERENCES**

1. Maskey D, Pradhan J, Kim HJ, Park KS, Ahn SC, et al. (2010) Immunohistochemical localization of calbindin D28-k, parvalbumin, and calretinin in the cerebellar cortex of the circling mouse. Neurosci Lett 483: 132-136.

2. Sarna JR, Hawkes R (2011) Patterned Purkinje cell loss in the ataxic sticky mouse. Eur J Neurosci 34: 79-86.

3. Jak M, van Bochove GG, Reits EA, Kallemeijn WW, Tromp JM, et al. (2011) CD40 stimulation sensitizes CLL cells to lysosomal cell death induction by type II anti-CD20 mAb GA101. Blood 118: 5178-5188.

4. Yilmaz M, Maass D, Tiwari N, Waldmeier L, Schmidt P, et al. (2011) Transcription factor Dlx2 protects from TGFbeta-induced cell-cycle arrest and apoptosis. Embo J 30: 4489-4499.

5. Lu WJ, Lee NP, Kaul SC, Lan F, Poon RT, et al. (2011) Mortalin-p53 interaction in cancer cells is stress dependent and constitutes a selective target for cancer therapy. Cell Death Differ 18: 1046-1056.

6. Williams C, Helguero L, Edvardsson K, Haldosen LA, Gustafsson JA (2009) Gene expression in murine mammary epithelial stem cell-like cells shows similarities to human breast cancer gene expression. Breast Cancer Res 11: R26.

7. Qin J, Suh JM, Kim BJ, Yu CT, Tanaka T, et al. (2007) The expression pattern of nuclear receptors during cerebellar development. Dev Dyn 236: 810-820.

8. Englund C, Fink A, Lau C, Pham D, Daza RA, et al. (2005) Pax6, Tbr2, and Tbr1 are expressed sequentially by radial glia, intermediate progenitor cells, and postmitotic neurons in developing neocortex. J Neurosci 25: 247-251.

9. Cheung CH, Lin WH, Hsu JT, Hour TC, Yeh TK, et al. (2011) BPR1K653, a novel Aurora kinase inhibitor, exhibits potent anti-proliferative activity in MDR1 (P-gp170)-mediated multidrug-resistant cancer cells. PLoS One 6: e23485.

10. Kim BJ, Zaveri HP, Shchelochkov OA, Yu Z, Hernandez-Garcia A, et al. (2013) An Allelic Series of Mice Reveals a Role for RERE in the Development of Multiple Organs Affected in Chromosome 1p36 Deletions. PLoS One 8: e57460.

11. Goetz JA, Singh S, Suber LM, Kull FJ, Robbins DJ (2006) A highly conserved amino-terminal region of sonic hedgehog is required for the formation of its freely diffusible multimeric form. J Biol Chem 281: 4087-4093.

12. Lin W, Metzakopian E, Mavromatakis YE, Gao N, Balaskas N, et al. (2009) Foxa1 and Foxa2 function both upstream of and cooperatively with Lmx1a and Lmx1b in a feedforward loop promoting mesodiencephalic dopaminergic neuron development. Dev Biol 333: 386-396.
